# Rare Coexistence: Pilocytic Astrocytoma With Atypical Teratoid/Rhabdoid Tumor Features in an Infant

**DOI:** 10.7759/cureus.55806

**Published:** 2024-03-08

**Authors:** Accha Nandini Sagar, Amar Taksande, Revat J Meshram

**Affiliations:** 1 Pediatrics, Jawaharlal Nehru Medical College, Datta Meghe Institute of Higher Education and Research, Wardha, IND

**Keywords:** treatment challenges, histological overlap, multidisciplinary management, atypical teratoid/rhabdoid tumor, pilocytic astrocytoma, pediatric brain tumors

## Abstract

This case report describes the presentation, diagnostic evaluation, and management challenges encountered in an eight-month-old female infant with fever, seizure, and a large cystic brain lesion initially diagnosed as pilocytic astrocytoma but later demonstrating atypical teratoid/rhabdoid tumor (AT/RT) features on histopathological examination-the infant presented with a fever and cold persisting for 10 days, followed by a seizure episode. Laboratory investigations revealed abnormalities, including anemia and leukocytosis. Imaging studies identified a large cystic lesion causing hydrocephalus. Despite initial treatment, the patient continued to experience seizures, prompting surgical intervention. Debulking surgery was performed, resulting in postoperative motor deficits. Subsequent imaging revealed persistent lesions, leading to further surgical intervention with shunt placement. Histopathological examination confirmed pilocytic astrocytoma with features suggestive of AT/RT. Despite counseling regarding poor prognosis and recommendations for chemotherapy, the parents declined further treatment, and the patient was discharged. This case underscores the diagnostic complexity and therapeutic dilemmas associated with rare histological overlaps in pediatric brain tumors, emphasizing the importance of multidisciplinary collaboration and tailored treatment strategies for optimal patient care.

## Introduction

Brain tumors represent a diverse group of neoplasms with significant variability in clinical presentation, histopathology, and treatment outcomes. In pediatric populations, brain tumors are the most common solid tumors and the leading cause of cancer-related morbidity and mortality [1]. Among pediatric brain tumors, pilocytic astrocytomas are frequently encountered and generally associated with a favorable prognosis [2]. Pilocytic astrocytomas are classified as World Health Organization (WHO) grade I tumors and typically present as well-circumscribed, slow-growing lesions, most commonly occurring in the cerebellum, optic pathways, and hypothalamus [3]. These tumors are characterized histologically by a biphasic pattern consisting of compacted bipolar cells and loose-textured regions containing Rosenthal fibers and eosinophilic granular bodies [4]. Despite their benign histology, pilocytic astrocytomas may manifest clinically with symptoms related to mass effects, such as headache, seizures, and focal neurological deficits [5].

In contrast, atypical teratoid/rhabdoid tumors (AT/RT) represent a highly malignant subtype of pediatric brain tumors, typically affecting infants and young children [6]. AT/RTs are characterized by aggressive growth patterns, early metastasis, and dismal prognosis, with most patients succumbing to the disease within a few months of diagnosis [7]. Histologically, AT/RTs exhibit rhabdoid cells with loss of INI1 (SMARCB1) expression, distinguishing them from other central nervous system neoplasms [8]. While pilocytic astrocytomas and AT/RTs represent distinct entities with differing biological behaviors, rare cases have been reported wherein these tumors exhibit overlapping features, posing diagnostic challenges [9]. Such cases may present with atypical clinical courses and variable treatment responses, necessitating a comprehensive approach to diagnosis and management.

In resource-limited settings, the diagnosis and management of pediatric brain tumors are further complicated by limited access to specialized diagnostic modalities, neurosurgical expertise, and oncological therapies [10]. Thus, optimizing outcomes in such settings requires judicious utilization of available resources and multidisciplinary collaboration among healthcare providers. The following case report describes the clinical presentation, diagnostic evaluation, and management challenges encountered in an infant with a rare presentation of pilocytic astrocytoma exhibiting features suggestive of AT/RT, highlighting the complexities associated with diagnosing and treating pediatric brain tumors in resource-limited settings.

## Case presentation

An eight-month-old female infant presented to a tertiary care hospital in central India with a chief complaint of fever and cold persisting for ten days, accompanied by a recent episode of seizure four days before admission. According to the mother's account, the child had been asymptomatic until ten days ago when she experienced a low-grade, intermittent fever that responded to medication. On Monday, the child was observed to be playful but subsequently had a seizure episode characterized by clenching of the mouth. She was initially taken to a government hospital, where she experienced another seizure associated with fever. The child displayed mouth clenching, hypertonia of all four limbs, and fist posturing, leading to referral to Amrawati for further evaluation and management.

**Figure 1:**
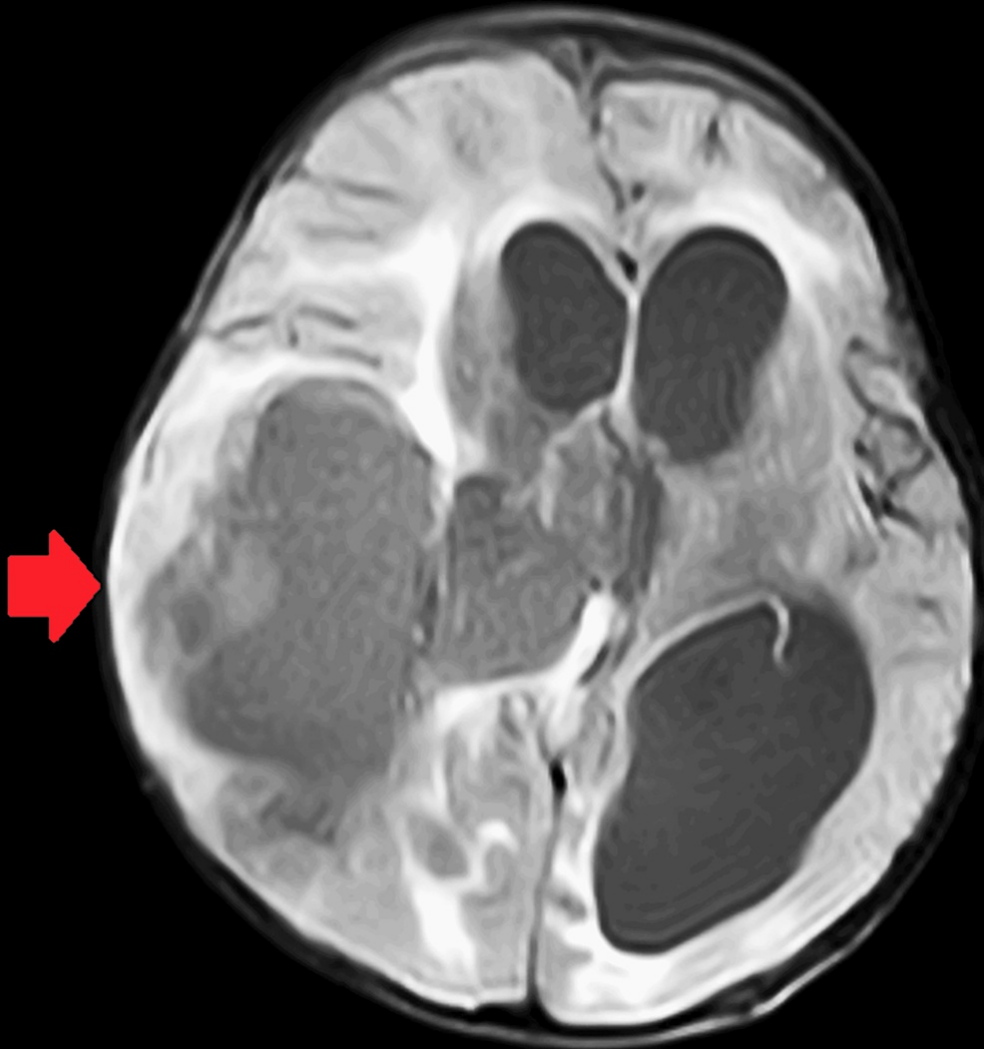
A large cystic lesion with an eccentric fluid-attenuated inversion recovery (FLAIR) heterogeneous component in the right fronto-parietal lobes, measuring 7.7 × 5.8 × 8.5 cm

Upon admission to a tertiary care hospital in central India, laboratory investigations revealed a hemoglobin level of 7.2 g/dL, total leukocyte count of 23,000/µL, platelet count of 361,000/µL, and C-reactive protein level of 30 mg/L. The coagulation profile showed abnormalities, although no bleeding manifestations were observed. The child received packed red blood cells and fresh frozen plasma transfusions, after which a repeat coagulation profile returned to normal. A plain MRI of the brain revealed a large cystic lesion with an eccentric fluid-attenuated inversion recovery (FLAIR) heterogeneous component in the right fronto-parietal lobes, measuring 7.7×5.8 × 8.5 cm, causing moderate to severe hydrocephalus and midline shift (Figure 1).

The differential diagnosis included pilocytic astrocytoma or pleomorphic xanthoastrocytoma. Moderate dilatation of the bilateral lateral and third ventricles with intraventricular hemorrhage was also noted. The child was initiated on intravenous ceftriaxone, pantoprazole, ondansetron, levetiracetam, and acetazolamide. Despite treatment, the child continued to experience multiple convulsive episodes, prompting the addition of phenytoin and later phenobarbital due to suspected raised intracranial pressure.

The child underwent debulking surgery for the suspected neoplastic mass, resulting in postoperative motor deficits on the right side. Following surgery, the child developed continuous fever spikes, necessitating antibiotic escalation to amikacin and metronidazole. Subsequent postoperative imaging revealed persistent cystic lesions and subdural collections, leading to further surgical intervention with the placement of a subdural subgaleal shunt.

**Figure 2:**
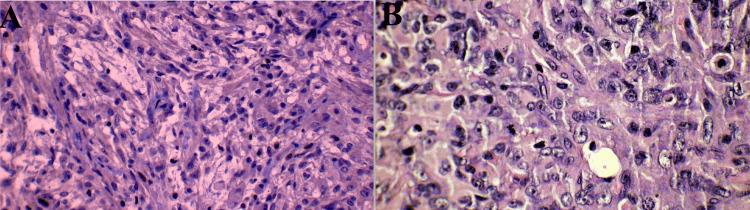
(A, B) Pilocytic astrocytoma, while immunohistochemical staining suggested an atypical teratoid/rhabdoid tumor, indicating a WHO grade 4 lesion

Histopathological examination confirmed the diagnosis of pilocytic astrocytoma, while immunohistochemical staining suggested an atypical teratoid/rhabdoid tumor, indicating a WHO grade 4 lesion (Figures 2A, 2B). Despite counseling from oncologists regarding the poor prognosis and recommendation for chemotherapy, the parents declined further treatment. Consequently, the child was discharged upon parental request.

## Discussion

The presented case highlights the diagnostic and therapeutic challenges associated with the coexistence of features from both pilocytic astrocytoma and AT/RT in pediatric patients. The coexistence of pilocytic astrocytoma and AT/RT features in our patient underscores the heterogeneity and complexity of pediatric brain tumors. A favorable prognosis typically characterizes pilocytic astrocytomas, while AT/RTs represent aggressive malignancies associated with poor outcomes [5,11]. The presence of AT/RT features within a seemingly benign tumor poses diagnostic challenges, as evidenced by the initial histopathological interpretation of pilocytic astrocytoma in our case. Accurate diagnosis relies on comprehensive histopathological evaluation, including immunohistochemical staining for specific markers such as INI1, typically lost in AT/RTs [8].

The management of pediatric brain tumors necessitates a multidisciplinary approach involving neurosurgery, oncology, radiation oncology, and supportive care services. In our case, the patient underwent surgical debulking followed by adjuvant therapy, including antiepileptic drugs and antibiotics for postoperative complications. However, despite aggressive intervention, the patient experienced disease progression and neurological deficits, highlighting the challenges in managing tumors with mixed histological features.

The prognosis in cases of pilocytic astrocytoma with AT/RT features remains uncertain, given the limited literature on such entities. While AT/RT features may present a worse outcome, the prognostic significance of this histological overlap requires further elucidation through more extensive case series and molecular profiling studies [12]. Additionally, the impact of treatment modalities, including chemotherapy and radiation therapy, on disease control and survival outcomes warrants investigation in this subset of patients.

Resource limitations pose significant barriers to optimal diagnosis and management of pediatric brain tumors, particularly in low- and middle-income countries [13]. Access to specialized diagnostic techniques, such as molecular profiling and advanced imaging modalities, may be limited in such settings, leading to diagnostic uncertainties and delays in treatment initiation. Moreover, comprehensive neuro-oncological care, including neurosurgical expertise and pediatric oncology services, may be scarce in resource-limited settings, compromising patient outcomes.

## Conclusions

In conclusion, the presented case highlights the intricate challenges encountered in diagnosing and managing pediatric brain tumors, particularly when faced with rare histological overlaps like the coexistence of pilocytic astrocytoma with features suggestive of AT/RT. Despite advancements in diagnostic techniques and therapeutic modalities, cases with mixed histological features remain diagnostically and therapeutically challenging, especially in resource-limited settings. Multidisciplinary collaboration among neurosurgeons, oncologists, pathologists, and supportive care teams is crucial for accurate diagnosis, treatment planning, and patient management. Continued research efforts to unravel the molecular mechanisms underlying these tumors and identify novel therapeutic targets are essential for improving outcomes and guiding personalized treatment strategies. Although the case underscores the complexities and uncertainties in managing rare pediatric brain tumors, it also emphasizes the importance of a comprehensive approach tailored to individual patient needs. By leveraging available resources, optimizing multidisciplinary care, and fostering collaboration among healthcare providers, we can strive to improve outcomes and enhance the quality of life for children affected by these challenging conditions. Ultimately, the management of pediatric brain tumors necessitates a holistic approach that addresses not only the biological characteristics of the tumor but also the psychosocial and supportive care needs of the patient and their family. Through collective efforts, we can continue to advance our understanding and management of these complex diseases, aiming for better outcomes and improved quality of life for pediatric patients with brain tumors.
